# Social identity and social integration: a meta-analysis exploring the relationship between social identity and social integration

**DOI:** 10.3389/fpsyg.2024.1361163

**Published:** 2024-04-04

**Authors:** Jieyi Hu, Chau Kiu Jacky Cheung

**Affiliations:** ^1^School of Humanities, Jinan University, Guangdong, China; ^2^Department of Social and Behavioral Sciences, City University of Hong Kong, Hong Kong SAR, China

**Keywords:** social identity, social integration, meta-analysis, quantitative research, social psychology

## Abstract

Social identity formation is crucial for psychosocial development, particularly in the case of migrating adults. A body of research exploring how social identity influences social integration among migrants shows that social identity affects social integration through a range of moderators and procedures. This study reports on a meta-analysis of 33 studies with 47 cases (total *N* = 33,777; Fisher’s *z* = 0.33, moderate effects) examining the relationship between social identity and social integration in research conducted from 2005–2020. The research findings suggest that social identity can affect social integration directly without any moderators, indicating that most of the identified moderators in the previous studies are sample-specific variables. More importantly, the effects of various aspects of identities exert similar degrees of impact (moderate effect) on social integration; in other words, the usefulness of analyzing different aspects of social identity on social integration is challenged.

In the last decade, immigration, a visible reflection of global integration related to well-being, freedom, life chances, etc., has become a significant facet of globalization, which has generated renewed interest in the rise of diverse identities. Social integration is recognized as the process of newcomers or minorities being incorporated into the host society (Alba and Nee, 1997) and is particularly significant within ethnic minority groups as they are trying hard to adapt to the host culture to function adequately daily and increase their well-being, which is the main benefit of social integration. Social integration refers to the extent of social relations or social ties binding people together in the sense of social belonging and inclusion (Wray et al., 2011). The effects of social integration have been well documented in the literature, including well-being (Scottham and Dias, 2010), self-esteem (Berry and Sabatier, 2010), health (Curran, 2003), social identity conflict (Ward et al., 2011), prosocial behaviors (Schwartz et al., 2007), inter-group relations (Zagefka and Brown, 2002), sociocultural adaptation problems (Neto et al., 2005), and objective integration parameters, such as income and occupation (Borjas, 1994). Individuals might also focus on the identity-affirming and acculturation strategies used in the host society in the face of demands linked to the context of immigration (Berry and Sam, 1997). Immigrants are more likely to be accepted if they come from cities or countries considered identically and culturally compatible with the host society (Wimmer and Soehl, 2014). At an individual level, the degree of identity assimilation into the host society results in social integration into the host culture (Helbling and Traunmüller, 2016). Social identity changes to adapt to the local environment as we embrace and occupy various spaces and form new ties under the conditions that identities are not fixed or given, which is exactly the process of social integration.

Scholars often postulate that social identity is linked to the process of social integration (Scott and Marshall, 2009). Social identity illustrates individual, interpersonal, and social processes to embed into the social structures (Davis et al., 2019). Social psychologists from both sociology and psychology have developed robust theories of social identity and social integration, which is notably represented by social identity theory. Social identity theory was proposed by Tajfel and Turner (1979) making a new interpretation of group behavior. An individual’s choice of behavior is largely affected by the perceived inter-group relationship, in that individual recognition of groups is the basis of group behavior (Tajfel and Turner, 1979). McCrone and Bechhofer (2008) and Reeskens and Wright (2013) suggested that social identity enacts identity meanings in social situations, which positively influenced social integration. Identities are internalized roles attached to the self as a unique person in a certain group (Burke and Stets, 2009; Stets and Serpe, 2013; Stets and Burke, 2014). Individuals tend to enact prominent identities when the social situation allows (Owens et al., 2010; Brenner et al., 2014). However, social identity may also hinder the process of social integration because of the strong ties those concerned have with their own countries (Aalberg et al., 2011). Previous literature has discussed diverse identities, such as ethnic social identity (Giles et al., 1979; Garcia, 1982; Moha, 2005), religious social identity (Walters et al., 2007), regional social identity (Moha, 2005) and cultural social identity (Ghosh, 2000). Given above, social identity is a multi-dimensional construct; thus, existing literature has examined the relationship between various aspects of social identity and social integration, while multiple moderators have been identified to influence the relationship between different identities and social integration. To illustrate, life satisfaction (Schotte et al., 2018), gender (Litchmore and Safdar, 2015), self-esteem (Vedder, 2005) and community involvement (Ramirez-Valles et al., 2010) tend to affect the relationship between social identity and social integration. Given such diverse results, the strength of relationship between different types of identities and social integration remains unknown. More importantly, it also remains unclear whether there is a need to separately examine how each of our identities influences social integration and identify those moderators for such a relationship. There is a huge variation in data collection within the existing literature, with data generated from different areas, ethnicities, and nationalities. Hence, a meta-analysis on the relationship of social identity and social integration is crucial and timely.

Social psychologists from both sociology and psychology also have developed acculturation theory besides social identity theory to explain social identity and social integration. Immigrant identities form through immigrants’ cultural and social positions in the host majority group, which performs integration or exclusion behaviors. The transition of migrants from one culture to another culture and/or from one society to another society may induce social identity challenges, which is analyzable by Berry’s (1990) acculturation model. The model focuses on the process of cultural exchange with another culture with general acculturative changes in the original culture (Berry, 1990), which can be summarized by four acculturation strategies (assimilation, separation, integration and marginalization). Furthermore, each acculturation strategy, effectively explaining the relationship between social identity and social integration, may lead to different psychological effects. For example, integration is the main characteristic of being a bicultural person and is the most commonly adopted acculturation strategy. Higher level of social identity increases immigrants’ social integration in the host countries through strong psychological linkage (Aalberg et al., 2011). Individuals organize identity meanings, religious affiliation, community connection, to respond to identity-relevant feedback, such as social integration (Walters et al., 2007) Existing literature on social identity offered two perspectives. One focused on the individual’s social identity and its relation to different social groups (Tajfel, 1982), while the second focused on how an individual cope with multiple identities and the results of this (Stryker and Burke, 2000). In addition, cross-cultural similarities are attributable to the integration tendency and the motivation of social integration and distinctiveness (Gaertner and Dovidio, 2000). Immigrants are expected to assimilate well into the local culture and quickly adopt a new social identity (Amit, 2012). The rapid development of technology and globalization effects has expanded the opportunities for people to acculturate (Chen et al., 2008; Wang and Abosag, 2019). In other words, people can acculturate because of immigration-or technology-mediated globalization. Both acculturation types may require people to integrate different cultural identities. Helbling and Traunmüller (2016) found that immigrants who shared the same race, ethnicity, culture or religion as the ethnic majority group were more likely to be accepted. This however, refutes the reality, given the large cultural distance particularly between East Asian culture and Western culture that migrants face in terms of social identity challenges. Existing psychology literature suggests that some ethnic minorities have developed integrated cultural identities whereas others have developed separated cultural identities (Haritatos and Benet-Martínez, 2002). The integrated cultural identities are more likely to lead to positive psychological well-being (Haritatos and Benet-Martínez, 2002).

The aim of our paper is to examine the relationship between social identity and social integration based on social identity theory and acculturation theory. The effects of moderators, life satisfaction (Schotte et al., 2018), gender (Litchmore and Safdar, 2015), self-esteem (Vedder, 2005) and community involvement (Ramirez-Valles et al., 2010), remained uncertain in the relationship between diverse identities and social integration. Meta-analysis is already widely used in psychology research; however, it is still relatively new in sociological studies, especially in social psychology research. To fill the research gap, our research presents meta-analysis endeavors to address two questions. First, we examine the average effect size of the relationship of social identity and social integration. Second, this paper examines the potential moderators between social identity and social integration.

## Literature review

### Social identity and social integration

“Social identity” firstly comes from psychology literature. As Freud (1921) suggested, social identity is the initial form of emotional connection with an objective object and it also implies an alternative to sexual instincts, as if it were that the object is injected into the self. One’s social identity includes a series of constituent multiple identities, and each sub-social identity differs from the others.

The impact of social norms on individual attitudes and behaviors is through one’s social identity (Brown, 2000). Multiple identities may lead to social identity conflict. There are four identities which are widely recognized and examined in both psychology and social science literature—these are ethnic social identity (Giles et al., 1979; Garcia, 1982; Moha, 2005), religious social identity (Walters et al., 2007), regional social identity (Moha, 2005) and cultural social identity (Ghosh, 2000). Ethnic social identity, like other identities, serves as a multidimensional construct, involving ethnic attitudes, knowledge, and social behaviors (Giles et al., 1979; Garcia, 1982). One’s religious social identity seems to influence immigrants’ adaptation to the host society; for example, Walters et al. (2007) found that the black community in Canada felt less Canadian because of their religious affiliation (Catholic or non-Catholic). Regional social identity includes national social identity and community social identity, among others. Investigated the case of Canada related to the measurement of national social identity, which could be deemed as the measurement of regional social identity. Cultural social identity is the social identity or belonging to the culture of a certain group, which is a part of self-perception relating to other types of social identity; for example, ethnic social identity and national social identity (Moha, 2005). Ghosh (2000) postulated that our experiences of the past and present social, cultural and economic relations seemed to influence how we define ourselves. Although these four identities have been the most extensively studied, they nevertheless differ significantly from one another. For example, regional social identity is mainly developed from regional classification. As different ethnicity relate to different religious identity, ethnic social identity and religious social identity share some characteristics. Additionally, ethnicity also relates to region; that is, region is sometimes divided based on ethnic groups.

Social integration may be affected by social identity in that the strong ties those concerned have with their own countries (Aalberg et al., 2011). Social integration is a result of allowing every member, particularly migrants, to feel part of the community and connected to the host society. Social integration takes place when minority groups from other regions are incorporated into mainstream society; however, such integration is a long-term process of adopting a shared system of meaning, language, culture and the like (Sherman et al., 2006). Social integration is also a multi-dimensional construct which may include economic integration, cultural integration and psychological integration. Social identity integration is different from social integration and only captures the compatibility between one’s multiple identities which are strongly associated with social roles. Individuals may perceive their cultural identities conflicted and separated and have difficulties in integrating them in a unified sense (Benet-Martínez et al., 2002; Benet-Martínez and Haritatos, 2005). However, social integration is a more comprehensive term which includes various aspects of integration into the host society. Therefore, social identity integration is only a dimension of social integration and cannot be perceived as equivalent to social integration.

There are no consistent terminologies that researchers use to examine the phenomenon of social integration. Social inclusion and social cohesion have been used as the proxy to measure social integration (e.g., Atkinson, 2002; Marlier, 2003; Atkinson et al., 2004; Cordier et al., 2017). Social integration embodies the implication of social inclusion and social cohesion at society, group or individual levels. To be more specific, social inclusion indicates a user-friendly term referring to individuals accessing limited resources to their benefit and returns (Oxoby, 2009) in terms of improving the ability, opportunity, and dignity of those disadvantaged based on their social identity (World Bank, 2013). Dayton-Johnson (2003) suggested that social cohesion can be a feature of society depending on social capital accumulation from the perspective of economics. Thus, social inclusion indicates the process of attaining certain rights, such as employment, adequate housing, medical care and education when people try to adapt to the host culture. A sense of belonging to a society or the opposite feelings of exclusion are often perceived as two important features of social cohesion. Social cohesion infers the desirable feature a society generating a sense of belonging within the person concerned; a sense of belonging to the community and society. The opposite, however, can also be the case when such feelings/features of social cohesion deteriorate (Schmeets and te Riele, 2014). As Bernard (1999) suggested, social cohesion is a hybrid mental construction to achieve a consensus in a social entity. There are six common dimensions of social cohesion; these are social relations (Friedkin, 2004), identification (Jenson, 2010), equality (Novy et al., 2012), orientation towards the common good (Green and Janmaat, 2011), objective and subjective quality of life (KfW Bankengruppe, 2010), and shared values (Botterman et al., 2012).

Several empirical studies have examined the topics related to social integration (Zhou et al., 2014; Mariyam and Jose, 2017; Negru-Subtirica et al., 2017). Social integration is generally measured by *objective* indicators and *subjective* indicators. Objective indicators refer to the situation of social security (e.g., medical insurance, etc.), housing, vocational training, working hours per day, and personal income. Subjective indicators refer to social identity, social status, willingness to relocate, acceptance of local cultural values and social satisfaction. In a similar vein, Giambona and Vassallo (2014) proposed that social inclusion (or social integration) could be estimated by four specific indicators, which are (i) the percentage of people at risk-of-poverty after migration, (ii) the proportion of severely materially deprived people, (iii) the percentage of people living with very low work intensity, and (iv) school dropout rates (Finn and Rock, 1997).

### The relationship between social identity and social integration

People encapsulate multiple identities. Existing literature has therefore examined the relationship between different aspects of social identity and social integration. Most scholars suggested that the perceived social identity, like national social identity, cultural social identity, and ethnic social identity, can greatly influence social integration. For example, national identity, a kind of social identity, is in the presence of other countries in the context of people to build a “country” belonging to the “sense of identity.” McCrone and Bechhofer (2008) argued that national social identity can be captured by social identity; which might eventually lead to social inclusion or social exclusion. Specifically, all individuals claim particular identities given their roles and the groups they belong to in the social structure, which implies that they search for positive distinctiveness in dealings with other groups, so as to increase permeability of group boundaries (McCrone and Bechhofer, 2008; Hogg, 2018). Social identity is passed down in the form of fixed repertoire by power systems, under which people negotiate and ultimately transform identities (McCrone, 2001). The degree of negotiated process influences the level of social integration. Bourdieu (1987) and Corneo and Jeanne (2010) argued that the relationship between career and people’s character traits (i.e., social identity) contributes to how individuals perceive themselves and influences how they are perceived by the rest of the society. The components of social integration come from various elements such as language, race, culture symbols, habits, geography and behaviors (Orgad, 2015). Reeskens and Wright (2013) demonstrated that social integration for immigrants was influenced by the different types of identities and different conditions in a community. Social identity can influence life chances insofar as being “one of us” in the society, while interest or other factors in inclusion and exclusion is an issue of how can people integrate in a society (McCrone and Bechhofer, 2008).

Nevertheless, the negative effect of social identity on social integration also exists (Reitz et al., 2009). Reitz et al. (2009) contended that social identity sometimes hindered the process of social integration—for example, the ethnic social identity showed a clear negative effect on citizenship acquisition and the willingness to integrate into the Canadian society for immigrants in Canada (Reitz et al., 2009). To be specific, according to social identity theory, individuals with negative or insecure identity cope with “jump ship” (social mobility) strategy, especially if group boundaries are sufficiently permeable to permit this (Brown, 2019). Once a relevant social identity is thus engaged, the adaptation is occurred. However, if migrants hold a very strong social identity with their own society, it will be very hard for them to accept the new culture and values in the host society (Aalberg et al., 2011). The two opposing opinions represent the diverse influence of social identity on social integration; however, the claim of positive effect occupies the dominant position.

Social identity theory and acculturation theory are widely used to explain the relationship of social identity and social integration in most of the literature. Based on social identity theory, identities have three bases: person, role, and group/social (Stets and Serpe, 2013) denoting one’s position within the broader social structure (Burke and Stets, 2009). Forging ties among these bases in social identity theory is a key step in constructing a general theory of the self (Stets and Burke, 2000; Stets and Serpe, 2013). Herein, social identities influence both immigrants and host societies, particularly those that are related to social system and cultural development such as ethnic social identity (Duroy, 2011). Moreover, the theoretical framework of social identity takes account of the heterogeneities which cause differences in social integration outcomes (Merve, 2017). For example, the cultural social identity assimilation process requires people to accept and integrate into the host culture practices. Immigrants identify and classify themselves into social categories in the host society; namely, social identity (e.g., religious social identity and regional social identity) based on the sense of belonging. The process of forming social relationships is also a process of social integration.

Acculturation theory is also used to explain the mechanism of how social identity influences social integration. Acculturation is a process that occurs when minority groups in a society meet another culture (Berry and Kim, 1988). The acculturation process may require ethnic minorities to adapt to different values, beliefs, and behaviors (e.g., language) of the dominant society (Ramirez, 2007). Berry (2005) presented four ways—assimilation, separation, integration and marginalization—to manage social identity in a multicultural environment, particularly for immigrants. Goodman (2012), Joppke (2007) and Sniderman and Hagendoorn (2007) claimed that the ethnic majority may perceive international immigrants as lacking commitment to assimilation. The marginalized feeling among international immigrants results in a vicious cycle of failed integration which is hard to break (Adida et al., 2014). Even among integrated individuals, people may have developed different levels of integration. Social integration may be improved through educational classes (e.g., language class, literacy class and employment training programs), cultural assimilation, and social norms acceptance (Billeke et al., 2015). Some migrants have developed integrated cultural identities, and others have not (Benet-Martínez et al., 2002). Personality traits, accent, appearance and dress may contribute to these differences.

This study examines the relationship between social identity and social integration with the extension of social identity theory and acculturation model, since, to the best of the authors’ knowledge, no comprehensive study including various samples in terms of area, ethnicity, nationality and others has been undertaken. We are specifically interested in seeing whether this association differs according to which aspect of social identity is considered. We are also interested in whether this moderation effect would differ across social identity dimensions.

## Methods

### Search strategies

Because target participants are not limited to one nationality, the current study includes papers from databases, such as PsycINFO, Pro-Quest, CNKI. Relevant studies with key words social identity (identification, belonging, acculturation, multiculturalism) and to integration (inclusion, cohesion, adaption, transformation, exclusion) were identified when searching these databases. We followed Mol and Bus’s (2011) analytic procedures to search relevant studies in social science research. We selected all possible materials from the past 15 years from 2005 to 2020.

### Inclusion criteria

The selected articles had to meet the following inclusion criteria: (a) not a single case study; (b) no opinion and non-empirical articles; (c) internal migration or international migration; and (d) reports social identity and social integration. The rest of the materials were closely reviewed using specific criteria described below.

First, the correlations, or means, or the percentage of variance (*R*^2^) in social integration accounted for by social identity, or standard deviations provided presented the association between social identity and social integration. These association could be transformed into a Fisher’s *z* effect size. All materials for controlling the potential impact of different writing systems in the relationship between social identity and social integration (García and Cain, 2014). We retrieved all (published or unpublished) articles, dissertations, and conference papers before the year of 2021.

Second, we treated multiple, independent samples within one article as separate studies (Mol and Bus, 2011). For each study, we tried to find a match on the following sample size, age, vocations, life satisfaction, community involvement, self-categorization, and cultural conformity. If data were absent from the original materials, we emailed the authors concerned for the required information.

Third, studies had to assess both social identity and social integration with objective and quantitative tasks. Meta-analysis incorporates the synthesized effect of related empirical studies into the analysis. This study is the aggregation of information leading to a higher statistical power on the relationship of social identity and social integration.

Fourth, if a composite measure of social integration (e.g., police effectiveness plus social integration; social system plus social inclusion; or cognitive of discrimination plus social cohesion) was used, the resulting correlation was not included because we wanted to examine how the relationship between social identity and social integration is affected by the methods used to measure the variables.

Fifth, the measures of social identity and social integration used to calculate the correlations had to be taken at the same time point and with a group of people who shared commonalities (i.e., all the people are migrants, internal migrants or international migrants at the beginning of their migration lives).

### Coding procedures

Studies were coded according to participants characteristics and the characteristics of the assessments were used to measure social identity and social integration. Two independent coders completed a standard coding scheme per study, comprising (a) year of publication, (b) sample size (c) participant’s location or nationality, (d) first three authors, (e) type of publication, (f) type of social identity, (g) type of migration, and (h) where the data came from. Two coders coded 75% of all studies included. The inter-coder agreement for both study characteristics and outcome variables ranged between 77 and 100% across meta-analyses, all discrepancies between coders were settled in discussion, and consensus scores were used.

### Moderators

For each study included in our analysis, we coded for the key moderators of the relationship of social identity and social integration. With respect to type of measurement, we divided the independent variable ‘social identity’ into four kinds of social identity—ethnic, cultural, regional, and religious—which four kinds most used in research.

### Overview of studies

The current study includes 33 articles with 47 cases which involved 33,777 participants in total. Most studies used a correlational design. All indicators come from the correlation index which includes regression analysis, correlation analysis and factor analysis (i.e., factor loading). In the current meta-analysis, 11 studies followed longitudinal research design and two studies applied experimental research design. Table 1 lists all details of the important features from the selected studies.

**Table 1:** Summary of included studies.

References	*N*	Country	Type of publication	Type of identity	Type of migration	Data obtained from	Fisher’s *z*	SE
Mariyam and Jose (2017)	41	India	Journal article	Cultural identity	N/A	A village	0.30	0.16
Zhou et al. (2014)	243	UK	Journal article	Cultural identity	Internal migration	School	0.22	0.07
Bisin et al. (2016)	1,559	UK	Journal article	Ethnic identity, religious identity	International migration	Country	0.34	0.03
Hagerman (2018)	2,000	Mexico	Thesis	Regional identity	Internal migration	State	0.41	0.02
Hagerman (2018)	2,000	Mexico	Thesis	Regional identity	Internal migration	State	0.26	0.02
Robinson (2006)	182	US	Journal article	Cultural identity	N/A	School	0.34	0.08
Wenzel (2000)	179	Germany	Journal article	Regional identity (national identity)	Internal migration	Country	0.26	0.08
Wenzel (2000)	179	Germany	Journal article	Regional identity (national identity)	Internal migration	country	0.35	0.08
Vedder (2005)	256	Turkish and Surinamese	Journal article	Ethnic identity	International migration	School	0.25	0.06
Mukherjee et al. (2012)	125	US	Journal article	Regional identity (national identity)	International migration	School	0.35	0.09
Mukherjee et al. (2012)	125	US	Journal article	Regional identity (national identity)	International migration	School	0.51	0.09
Mukherjee et al. (2012)	125	US	Journal article	Regional identity (national identity)	International migration	School	0.23	0.09
Rydell et al. (2010)	185	US	Journal article	Ethnic identity	International migration	School	0.37	0.07
Maxwell (2017)	2,396	France, Germany & US	Journal article	Regional identity (national identity)	International migration	Three countries	0.29	0.02
Litchmore and Safdar (2015)	77	Canada	Journal article	Ethnic identity	International migration	State	0.23	0.12
Bradford (2014)	1,017	UK	Journal article	Regional identity (national identity)	International migration	City	0.32	0.03
Brigevich (2016)	8,347	France	Journal article	Regional identity, cultural identity	International migration	Country	0.34	0.01
Brigevich (2016)	8,347	France	Journal article	Regional identity, cultural identity	International migration	Country	0.35	0.01
Ramirez-Valles et al. (2010)	643	US	Journal article	N/A	N/A	Cities	0.30	0.04
Ramirez-Valles et al. (2010)	643	US	Journal article	N/A	N/A	Cities	0.23	0.04
Amit (2012)	1,000	Israel	Journal article	Regional identity	International migration	Countries	0.29	0.03
Amit (2012)	1,000	Israel	Journal article	Regional identity	International migration	countries	0.31	0.03
Maliepaard and Phalet (2012)	2,027	Netherlands	Journal article	Religious identity	International migration	Country	0.31	0.02
Lapalme and Doucet (2018)	290	Canada	Journal article	Cultural identity	N/A	Three agencies	0.32	0.06
Reid (2013)	190	US	Journal article	Ethnic identity	International migration	School	0.28	0.07
Passini (2013)	192	Italy	Journal article	Cultural identity	N/A	Country	0.32	0.07
Kuo et al. (2017)	228	US	Journal article	Ethnic identity	International migration	Country	0.33	0.07
Carmeli and Shteigman (2010)	150	Israel	Journal article	Cultural identity	N/A	Firms	0.32	0.08
Schilpzand and Huang (2018)	733	China	Journal article	Cultural identity	N/A	Firms	0.30	0.04
Schilpzand and Huang (2018)	733	China	Journal article	Cultural identity	N/A	Firms	0.30	0.04
Guo et al. (2015)	342	China	Journal article	Cultural identity	Internal migration	City	0.36	0.05
Chu et al. (2012)	1,230	China	Journal article	Regional identity	Internal migration	City	0.35	0.03
Luo and Lu (2013)	284	China	Journal article	Cultural identity	Internal migration	City	0.35	0.06
Luo and Lu (2013)	284	China	Journal article	Cultural identity	Internal migration	City	0.36	0.06
Luo (2014)	547	China	Journal article	Regional identity	Internal migration	Three cities	0.32	0.04
Luo (2014)	547	China	Journal article	Regional identity	Internal migration	Three cities	0.38	0.04
Luo (2014)	547	China	Journal article	Regional identity	Internal migration	Three cities	0.28	0.04
Yang (2015)	533	China	Journal article	National identity & ethnic identity	Internal migration	Three cities	0.32	0.04
Yang (2015)	533	China	Journal article	National identity & ethnic identity	Internal migration	Three cities	0.38	0.04
Wang et al. (2013)	1,550	China	Journal article	Cultural identity & regional identity	Internal migration	City	0.39	0.03
Wang et al. (2013)	1,550	China	Journal article	Cultural identity & regional identity	Internal migration	City	0.32	0.03
Zhao (2016)	1,340	China	Thesis	Ethnic identity	Internal migration	Province	0.33	0.03
Zhao (2016)	1,340	China	Thesis	Ethnic identity	Internal migration	Province	0.37	0.03
Wang and Xu (2013)	3,994	China	Journal article	Regional identity	Internal migration	Province	0.33	0.02
Cui (2012)	1,171	China	Journal article	Regional identity	Internal migration	City	0.30	0.03
Fu (2016)	335	China	Journal article	Regional identity & cultural identity	Internal migration	City	0.51	0.06
Huang (2011)	391	China	Thesis	Regional identity	Internal migration	School	0.27	0.05

### Meta-analytic procedures

The Comprehensive Meta-Analysis 3.0 software was applied to Fisher’s *z-*effect sizes calculation. PRISMA was applied to the current method, which provided the detailed information for a meta-analysis (Moher et al., 2010). All correlations between the social identity and any outcome variable were inserted into the computer program Comprehensive Meta-Analysis and transformed into Fisher’s *z-*effect sizes for further analyses, because the variance of Fisher’s *z* was nearly constant, whereas the variance of the correlation followed an asymmetrical distribution (Borenstein et al., 2009). To ease interpretation of the Results section, Fisher’s *z* summary estimates were transformed back into a correlation with the formula *r* = tanh(*z*’) (Lipsey and Wilson, 2001). In general, a Fisher’s *z* value of 0.10 (*r* = 0.10) counted as a small-effect size, 0.31 (*r* = 0.30) as moderate, and 0.55 (*r* = 0.50) as large (Cohen, 1988).

To interpret the ES for the correlation between social integration and its domains (economics, psychological belonging, cultural acceptance, etc.), we regraded each outcome domain as an independent correlate (Mol and Bus, 2011). If an article applied multiple tests to measure the correlation through different measurements, we reported the average ES to ensure each study only offers one ES in the analysis.

We coded samples as “ethnic” when the article offered the relevant information (i.e., language, history and common social norms). In the current study, we treat participants in the same way as those who live in one area with local people because they share similar social norms of the host society.

The current study applied a random-effect model for conservative consideration on ES estimation (Borenstein et al., 2009). We also presented 95% confidence interval (CI) and ensured the interval did not cross the zero zone (Pigott, 2012). Meanwhile, we presented the chi-square test for materials’ heterogeneity report; we selected a significant *Q* between the df value for moderator analyses (meta-regression would apply if the minimum number of cases exceeds four) (Mol and Bus, 2011).

Because the impact of social identity and its domains (ethnic social identity, cultural social identity, regional social identity and religious social identity), studies in each domains showed different correlations. Therefore, we examined whether the results were moderated by the age, vocation, life satisfaction, community involvement, self-categorization, and cultural conformity, among other factors. As another indicator, we checked the publication bias. We applied funnel plot (Duval and Tweedie, 2000), *p*-curve analysis (Simonsohn et al., 2014) and PET-PEESE6 (Stanley and Doucouliagos, 2014). Last, we presented Rosenthal’s fail-safe number which reveals the number of missing studies with null effects that would have to be retrieved and included in the analyses before the *p* value becomes non-significant (Borenstein et al., 2009).

## Results

### Overall analyses

Table 2 presents the overall effect of the relationship of social identity and social integration. From the table, the overall effect between different kinds of social identity and social integration is at a moderate level (Fisher’s *z* = 0.33, *p* < 0.001). The chi-square test shows that the differences in the indicators of the empirical studies involved in this research were not significant (*p* > 0.05), which means that the heterogeneity had not reached a level of significance. The results show that the correlation between all kinds of social identity and social integration were at the moderate level, which also indicates that almost all potential moderators are not significant in this correlation. As for the heterogeneity analysis, we test all kinds of moderators. The results show that these moderators (e.g., ethnic group, age group) only explain 8% of the variance of the heterogeneity; 92% of the variances of the heterogeneity are not explainable by the current moderators.

**Table 2:** Meta-analysis of the correlation between social identity and social integration.

	k	Sample N	Fisher’s *z*	95%CI	Q	I^2^	Fail-safe N
Social identity—social integration	43	37,671	0.33^***^	[0.32, 0.34]	48.34	13.11	16,214

^***^p < 0.001.

**Figure 1:**
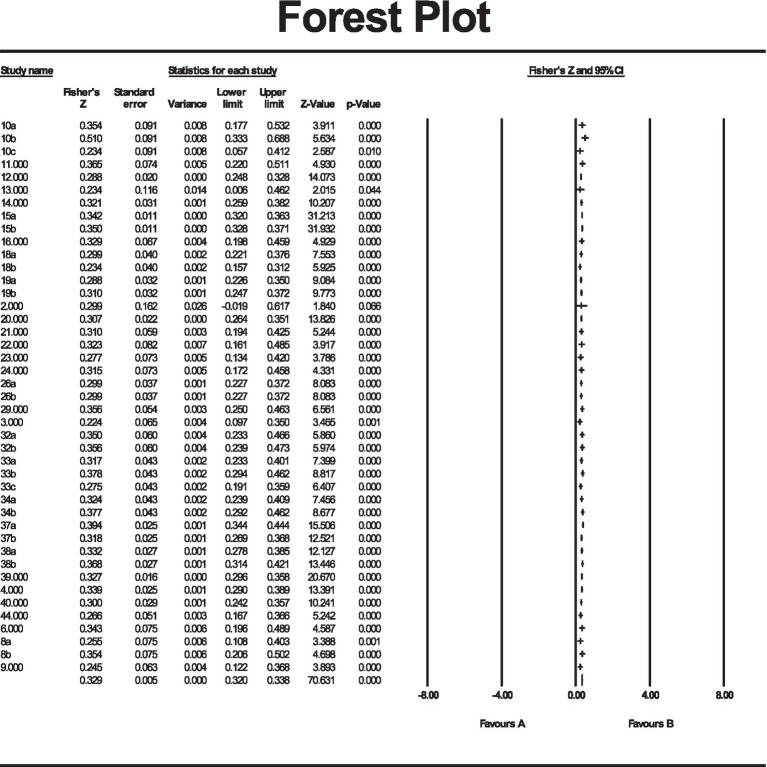
Forest plot of precision by Fisher’s *z* of the effect sizes relating to social identity and social integration.

The forest plot in Figure 1 shows the aggregate raw proportions and the 95% CIs for all the studies included in the meta-analysis. Figure 1 presents the ES and 95% CIs from all studies included in this research. The figure shows the overall ES at the bottom from the selected studies, and the dotted line at the 0.50 level reflects that the selected materials are randomly distributed with no researcher preferences.

In the absence of publication bias we would expect the studies to be distributed symmetrically about the combined effect size. By contrast, in the presence of bias, we would expect that the bottom of the plot would show a higher concentration of studies on one side of the mean than on the other. This would reflect the fact that smaller studies (which appear toward the bottom) are more likely to be published if they have larger-than-average effects, which makes them more likely to meet the criterion for statistical significance.

Various statistical procedures can be accessed from the View menu to quantify or augment this display. The classic fail-Safe N and the Orwin fail-safe N ask if we need to be concerned that the entire observed effect may be an artifact of bias. Rank correlation and regression procedures can test for the presence of bias. Trim and fill analysis offers a more nuanced perspective, and asks how the effect size would shift if the apparent bias is to be removed.

An important caveat is confirmed by Sterne and Egger (2001), which notes that while the plot and these procedures may detect a relationship between sample size and effect size, they cannot assign a causal mechanism to it.

Prior to exploring the effect of moderators found in the literature, however, we examined to what extent the overall effect may be influenced by publication bias.

### Publication bias check

**Figure 2:**
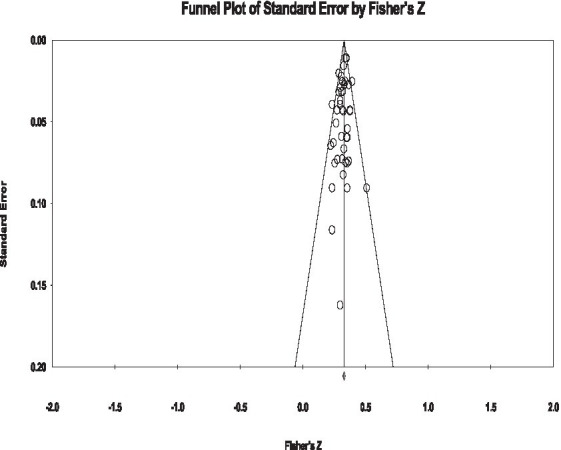
Funnel plot of the effect sizes relating to social identity and social integration.

The funnel plot distributes the large SE studies across the top of the plot which shows the selected studies are of a high quality. Regarding the current study, most studies are dotted across the top of the funnel plot, which means the selected materials are of a higher quality. Also, from the plot (see Figure 2), we see that almost all studies are distributed randomly axisymmetric, which reflects that publication bias was not significant.

Then we use Egger’s regression intercept test which was suggested by Sterne and Egger (2001) to offer statistical cues to test the publication bias. From the conservative perspective view, we focus on the random effects model and test the asymmetry of the funnel plot distribution. The correlation rank test from Begg and Mazumdar (1994) showed that two indicators did not reach a level of significance (*p* > 0.05), which means that from the statistical perspective, the publication bias was not significant.

We test the estimate indicator credibility through *p*-curve analysis (Simonsohn et al., 2014). Both “file drawer effect” and “just significant effects” evinced that the obtained effect is strong and the sensitivity analysis is not significant (*p* > 0.50). It also shows that the “right-skewed curve” is not significant (*z* = 0.03, *p* > 0.05), and that the “left-skewed curve” also do not reach a level of significance (*p* > 0.05). In sum, the results of *p*-curve analysis demonstrate the current study do not have significant publication bias.

Based on PET-PEESE method checking, because our results are significant (*r* = 0.32, *SE* < 0.01, *p* < 0.001), either the fixed model or the random model shows similar results (i.e., *r* = 0.32, 95% CI [0.31, 0.33], *p* < 0.001). According to Stanley and Doucouliagos’s (2014) findings, the current study do not find significant publication bias.

Regarding the safe-N number, Table 1 shows that 16,214 samples are still needed to reverse the conclusion reached that the correlation is not significant. Also, the result is much more stable and the researcher preference factor is not significant. In terms of checking the unpublished studies, *r* = 0.31, 95% CI [0.30, 0.32]. This reveals a marginal difference in favor of published studies (*Q* = 48.34; *df* = 41, *p* = 0.23). Since they share a similar ES with published studies, the overlapping CI achieves the level of legitimacy. In sum, the safe-N number analysis show that the current study do not have significant publication bias.

### Moderation analyses

Unlike other meta-analysis research (Lee and Ahn, 2013; Thielmann et al., 2020), this meta-analysis on the relationship of social identity and social integration (see Table 3) suggests that all the moderators in previous literature show weak influences on the relationship between social identity and social integration. Possibly, there is no need to find moderators for the relationship. The effect of social identity on social integration is robust to moderation. Community involvement (Ramirez-Valles et al., 2010), life satisfaction (Schotte et al., 2018), self-esteem (Vedder, 2005) and gender (Litchmore and Safdar, 2015) do not seem to demonstrate moderation effects (see Table 3).

**Table 3:** Moderation effect between social identity and social integration.

	Mean	SD	*F*	*p*-value
Ethnic identity	0.28	0.05		
Cultural identity	0.31	0.11		
Regional identity	0.26	0.19		
Religious identity	0.29	0.13		
			0.39	0.76

^***^p < 0.001.

## Discussion

The research findings (shown in Table 2) suggests that various social identity types, such as ethnic social identity, religion social identity, regional social identity and cultural social identity classified by existing literature, exert similar degrees of impact on social integration; namely, around the moderate effect—Fisher’s *z* = 0.33. In other words, the influences of social identity on social integration is the same regardless of social identity types. This also suggests that the classification of social identity is not necessary since any classifications have a similar impact on social integration. There is no strong evidence to support the research which examines the influences of each type of social identity on social integration separately. Different types of social identity correlate with each other; in other words, it is hard to distinguish which one influences social integration without affecting other identities. For example, cultural social identity is a sense of belonging which in turn affects other identities such as ethnic social identity and national social identity (Moha, 2005). Litchmore and Safdar (2015) attempted to distinguish religious social identity and ethnic social identity, but religious social identity usually affects ethnicity. More importantly, none of the moderators significantly influences the relationship between social identity and social integration, including community involvement (Ramirez-Valles et al., 2010), life satisfaction (Schotte et al., 2018) and self-esteem (Vedder, 2005) that exists in the selected studies. The findings suggest that social identity and social integration have a stronger relationship without any moderators, which may indicate that moderators are context-specific and only suitable for a certain sample. The relationship between social identity and social integration cannot follow an exclusionary pattern (Tatar, 2010). This meta-analysis provided convincing evidence for the direct relationship between social identity and social integration rather than occurring through moderators; therefore, the need for future research to identify the potential moderators between social identity and social integration should be cautious.

The research findings generate some practical implications. First, the governments of those multicultural societies (e.g., the US and the UK) should try to create opportunities for migrants to integrate into the mainstream culture in general without identifying which specific social identity the governments should focus on. For instance, the host government could offer language classes for migrants to learn the local language, which is generally deemed as increasing cultural social identity in the previous literature. The increasing cultural social identity of migrants will probably enhance their national social identity and other identities as well. Eventually, social integration among migrants will be improved; this is also the process to help migrants to adapt to the host environment. For the migrants, participating in and interacting with the host country could be a process that may lead to social integration. During this process, their levels of social identity increase when they participate in the local activities.

## Limitations

This meta-analysis is not without limitations. First, the number of studies involved is limited, which may affect the results of the meta-analysis. Although we have searched almost all the literature between 2005 and 2020, literature that meet our selection requirement is limited. Eventually, 33 studies with a total number of 33,777 cases were selected. Similarly, this meta-analysis has involved 15 years of studies from 2005 to 2020. Future researchers may consider expanding the time frame and analyzing studies published before 2005 or after 2020. Second, only 8% of the variance on heterogeneity was explained, so other moderators which may explain the relationship between social identity and social integration need further exploration. Finally, improvements could not be made to the identified effects due to the lack of psychometric data. Some psychological factors may affect social identity, and then further influence the relationship between social identity and social integration. This limitation offers suggestions for future researchers that relevant psychometric data should be considered and collected.

## Conclusion

This meta-analysis serves as the first study to provide a stable estimation of the relationship between social identity and social integration based on previous research (around from 2005 to 2020) with territorial (studies from all over the world), and numerical (34 independent studies, moderate effect, and the total sample number of 33,777) factors. This meta-analysis creatively focuses on social identity and social integration, which is one of only a few meta-analysis studies in psychosocial literature. Indeed, multilevel models provide more robust estimations for the observed effects and the effects of moderators. Although social identity has a strong connection with social integration, this study illustrates different types of social identity exert similar influence on social integration. Herein, there is not necessary to classify social identity. Moreover, this study also suggests that the moderators of the relationship between social identity and social integration are only suitable for particular samples. Findings on potential moderators between social identity and social integration are not yet generalizable.

## Data availability statement

The original contributions presented in the study are included in the article/supplementary material, further inquiries can be directed to the corresponding author.

## Author contributions

JH: Writing – original draft. CC: Writing – review & editing.
